# Effects of warm needling therapy on symptoms of benign prostatic hyperplasia: A protocol for a systematic review and meta-analysis

**DOI:** 10.1097/MD.0000000000028038

**Published:** 2021-12-03

**Authors:** Tao Zhang, Bin Li, Hui-Lin Liu, Shao-Song Wang, Fan Zhang, Xin Du, Wei You, Lian-Cheng Jia, Jing-Qing Sun

**Affiliations:** a Department of Acupuncture and Moxibustion, Beijing Hospital of Traditional Chinese Medicine, Capital Medical University, Beijing Key Laboratory of Acupuncture Neuromodulation, Beijing, China; b Department of Urology, Beijing Hospital of Traditional Chinese Medicine, Capital Medical University, Beijing, China.

**Keywords:** benign prostatic hyperplasia, effect, meta-analysis, protocol, warm needling

## Abstract

**Background:**

Benign prostatic hyperplasia (BPH) is the term for a type of non-malignant prostate enlargement that is most often diagnosed in men of middle age and older. Lower urinary tract symptoms (LUTS) are commonly observed in men afflicted with BPH. Evidence suggests that warm needling therapy could be applied clinically to relieve the LUTS associated with BPH, particularly in China, where experienced practitioners are readily available. In this review, the safety and effects of warm needling therapy are assessed in the context of treatment for LUTS associated with BPH.

**Methods:**

First, data for relevant randomised controlled trials and the initial periods of randomised cross-over trials will be obtained from four English databases (MEDLINE, Cochrane Central Register of Controlled Trials, EMBASE, and Allied and Complementary Medicine Database) and six Chinese databases (China National Knowledge Infrastructure, Wanfang Database, SinoMed, Chongqing VIP Chinese Science and Technology Periodical Database, China Master's Theses Full-text Database and China Doctoral Dissertations Full-text Database). The primary outcomes analysed in this protocol are improvements in urological symptoms as measured by recognized urological symptom scores, while secondary outcomes include improvement of urine flow rate measures, residual urine volume, nocturia, prostate size, and quality-of-life score. In addition, safety outcomes will be analysed by assessing incidences of adverse events. Two reviewers will independently assess and select studies, extract data and assess the risk of bias. Data synthesis and risk bias assessment will be performed with Review Manager software (version 5.3).

**Results:**

This systematic review provides a synthesis to assess the therapeutic efficacy of warm needling therapy for LUTS associated with BPH.

**Conclusion:**

The present study will provide a clinically relevant evaluation of the current state of evidence regarding the therapeutic efficacy of warm needling therapy for LUTS associated with BPH.

**Ethics and dissemination:**

Ethical approval is not required for this review, because private information will not be collected from the included participants. The results of the study will be published in a peer-reviewed journal.

**Registration number:**

PROSPERO CRD42020198360.

## 1 Introduction

Benign prostatic hyperplasia (BPH) refers to the clinically relevant proliferation of smooth muscle cells and epithelial cells in the prostatic transition region, which commonly occurs in men of middle age and older.^[1,2]^ Nearly half of patients afflicted with BPH experience lower urinary tract symptoms (LUTS), which are characterized by storage, voiding, and postmicturition symptoms, including hesitancy, poor stream, increased frequency, increased urgency, and nocturia.^[3]^ As men age, the prevalence of LUTS increases in a closely related manner.^[4]^ BPH occurs most commonly in males over the age of 40.^[5]^ The prevalence of BPH is approximately 25% to 40% in males in their 50 s, 50% in males in their 60 s, and more than 75% in males in their 80 s.^[5–8]^ Evidence suggests that the prevalence of BPH in Chinese patients is similar to that of patients in Europe and North America.^[9]^ A recent study reported that the prevalence of BPH was roughly 47.0% in men older than 60 who were outpatients of urology departments in China.^[10]^ Intriguingly, the symptoms experienced by Chinese patients afflicted with BPH tended to be more severe than those of BPH patients from the United States.^[11]^ Although BPH is non-carcinogenic, the condition has a profound effect on the quality of life, physical health and psychological state of patients afflicted with it.^[9]^ Moreover, BPH is associated with increased health care costs due to the necessity of treating the associated LUTS to restore patient health and quality of life.

Treatments for BPH include conservative, pharmacological, and surgical therapy.^[12]^ Conservative therapy, which includes watchful waiting and modification of behavior and diet, is recommended as a first treatment step for patients with mild or moderate BPH. Pharmacological therapy is recommended as a subsequent treatment step according to the characteristics of particular patients, including prostate size, level of prostate-specific antigen (PSA), and simultaneous phenomena. Unfortunately, patient compliance with regard to administration of pharmacological therapies is limited by the associated side effects and the use of drug combinations for the presence of underlying diseases. Therefore, surgical treatment is recommended for patients with poor compliance to pharmacological therapy and those with related complications.

Warm needling therapy is a traditional Chinese medicinal practice that has been applied widely in China and the surrounding region for millennia.^[13]^ To perform warm needling therapy, an acupuncture needle is warmed by placing an ignited moxa stick (composed of dried mugwort) in contact with the handle of the needle after the needle is inserted into the patient.^[14]^ This practice imparts acupuncture stimulation via needling, heat stimulation via infrared radiation, and pharmacological action via the use of dried mugwort.^[15,16]^ Warm needling therapy combines the therapeutic effects of acupuncture and moxibustion, and it has been shown to be safe in clinical practice.^[17]^ The practice of warm needling therapy can be used to influence a multi-dimensional physiological network composed of the circulatory system, nervous system, endocrine system, and immune system, and thus achieve the clinical goals of maintaining physiological homeostasis and treating diseases.^[18]^ The benefits and safety of warm needling therapy as a treatment for LUTS associated with BPH have been explored in clinical trials, which revealed that it had a significant impact on the disease. However, the relationship between warm needling therapy and BPH has not been subjected to a comprehensive statistical analysis. Therefore, the aim of this study is to collect evidence and evaluate the effect and safety of warm needling therapy for LUTS associated with BPH.

## 2 Methods

### 2.1 Criteria for study inclusion

#### 2.1.1 Types of studies

Randomized controlled trials (RCTs) and the first periods of randomized cross-over trials will be included, regardless of their publication status. Duplicate publications and trials with incomparable baselines will not be subjected to analysis.

#### 2.1.2 Types of participants

Patients with BPH diagnosed using standardized diagnostic criteria will be included in the analysis. The following individuals will be excluded from the analysis: individuals with serious neurologic, neurodegenerative, psychiatric, or systemic disorders; individuals with other diseases associated with urinary tract symptoms, including neurogenic bladder cancer and prostatic cancer; and individuals who are allergic to acupuncture or moxibustion. The enrolled participants will not be restricted with regard to ethnicity, age, or economic status.

#### 2.1.3 Types of interventions

The treatment group will include patients who underwent warm needling therapy alone or in combination with other conservative treatments, such as acupuncture, moxibustion, or pharmacological treatments. However, patients who underwent combination therapy with three or more interventions or interventions with potential safety hazards will be excluded. The control group will include patients who underwent no treatment or were placed on a waiting list, as well as those who were treated with sham warm needling or pharmacological treatments.

#### 2.1.4 Types of outcomes

The primary outcome for the analysis will be changes in urological symptoms as measured by validated urological symptom scores, including but not limited to the International Prostate Symptom Score (IPSS), the American Urologic Association Symptom Score, and the Boyarsky Score.

The secondary outcomes for the analysis will include improvements in urine flow rate measures, residual urine volume, nocturia, prostate size, and quality-of-life score. In addition, incidences of adverse events, including scalding, coughing, allergy, bleeding during or after needling, breaking or winding of needles, fainting, and local infection.

### 2.2 Study selection methods

Four English databases and 6 Chinese databases will be searched for evidence dating from their inception until December 2021: MEDLINE via PubMed, the Cochrane Central Register of Controlled Trials (CENTRAL), EMBASE via Elsevier, the Allied and Complementary Medicine Database (AMED) via EBSCO, China National Knowledge Infrastructure (CNKI), Wanfang Database, SinoMed, the Chinese Scientific Journal Database (VIP), the China Doctoral Dissertations Full-text Database (CDFD), and the China Master's Theses Full-text Database (CMFD). The databased will be searched using the following medical search headings (MeSH): benign prostatic hyperplasia, benign prostatic hypertrophy, warm needling, warm needle, warm acupuncture, randomised controlled trial, controlled trial, randomised, randomly, clinical trial, and comparative study. Chinese databases will be searched using translations of these search terms agreed upon by 2 independent reviewers, who will be fluent speakers of Chinese. Table 1 summarizes the search strategy that will be used for MEDLINE. The search strategies for the other databases used for this analysis will be determined according to the individual requirements of each database.

**Table 1:** MEDLINE search strategy.

Number	Search item
#1	randomised controlled trial
#2	controlled trial
#3	comparative study
#4	randomised
#5	randomized
#6	randomly
#7	trial
#8	#1 OR #2 OR #3 OR #4 OR #5 OR #6 OR #7
#9	benign prostatic hyperplasia
#10	benign prostatic hypertrophy
#11	prostatic hyperplasia
#12	prostatic hypertrophy
#13	#9 OR #10 OR #11 OR #12
#14	warm needling
#15	warm needle
#16	warm acupuncture
#17	#14 OR #15 OR #16
#18	#8 AND #13 AND #17
#19	remove duplicates from #18

### 2.3 Data collection and analysis

#### 2.3.1 Study selection

**Figure 1:**
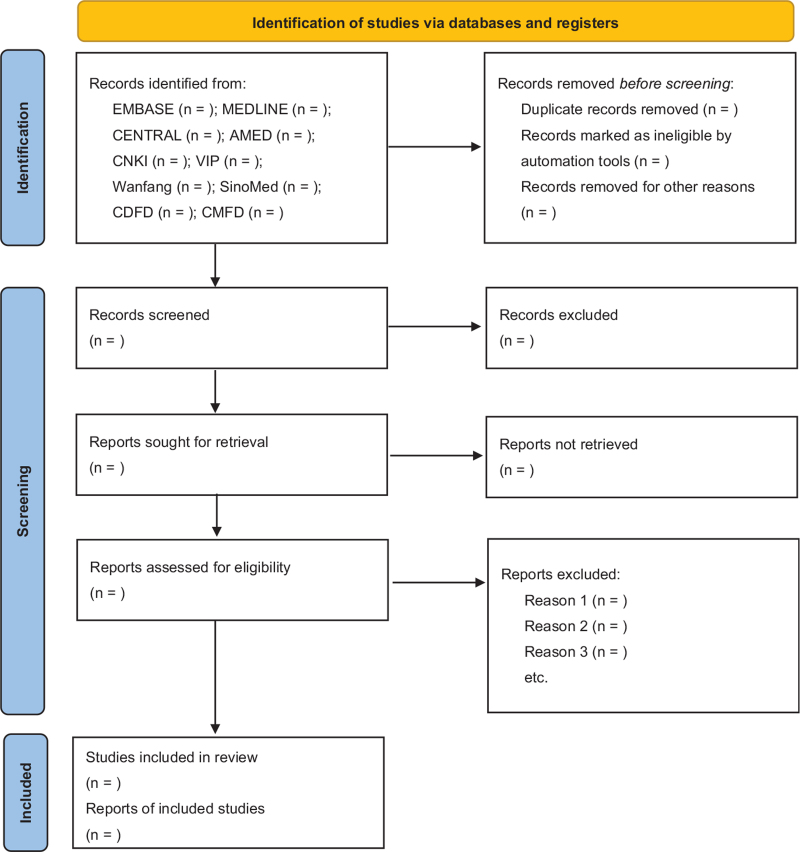
PRISMA 2020 flow diagram of the analysis.^[22]^ AMED = Allied and Complementary Medicine Database, CDFD = China Doctoral Dissertations Full-text Database, CENTRAL = Cochrane Central Register of Controlled Trials, CMFD = China Master's Theses Full-text Database, CNKI = China National Knowledge Infrastructure, PRISMA = Preferred Reporting Items for Systematic Review and Meta-Analysis, VIP = Chinese Scientific Journal Database.

Two independent reviewers, SSW and FZ, will identify and screen clinical studies for inclusion in the analysis. First, the reviewers will screen the titles and abstracts. If the reviewers cannot confirm whether or not the inclusion criteria are met by the studies based solely on the abstracts, then the full texts will be read accordingly. Any trials that meet the inclusion criteria for the analysis will be collected and cross-checked by the 2 reviewers mentioned above. A third reviewer, BL, will be included in the selection process for discussion of any dissimilarities in the pool of selected studies, after which BL will cross-check the studies in question and make a final decision regarding whether they should be included in the analysis. The study selection process is depicted in a PRISMA 2020 flow diagram in Figure 1.

#### 2.3.2 Data extraction and management

Two independent reviewers, XD and LCJ, will extract the data from the selected studies and complete a standardized data extraction form, which will include information about the sample size, interventions, treatment frequency, treatment course, outcomes, and adverse events. XD and LCJ will cross-check the resulting dataset, after which any disagreements regarding data extraction and management will be discussed with a third reviewer, HLL, who will make a final decision on the matter.

#### 2.3.3 Risk of bias assessment

The risk of bias of the included studies will be assessed by 2 independent reviewers, TZ and JQS, using the “Risk of Bias” tool described in the Cochrane Handbook (version 5.1). The types of bias evaluated by the selected assessment process include selection bias (randomization and concealment of allocation), performance bias (blinding of practitioners and participants), detection bias (blinding to outcome assessment), attrition bias (incomplete datasets and participant dropout), and reporting bias (selective reporting).^[19,20]^ TZ and JQS will cross-check the results of the assessment, and any disagreements regarding the risk of bias assessment will be discussed with a third reviewer, BL, who will make a final decision on the matter.

#### 2.3.4 Measures of treatment effect

Review Manager statistical software (RevMan, version 5.3) will be used for all data analysis. A risk ratio (RR) with 95% confidence intervals (CIs) will be used for the analysis of categorical variables, whereas continuous variables will be subjected to analysis of mean difference (MD) with 95% CIs.

#### 2.3.5 Unit of analysis issues

Before the statistical analysis, the units of the outcomes from each of the selected studies will be converted into the International System of Units.

#### 2.3.6 Procedures for missing data

Missing or inadequate data will be obtained from the first or corresponding author by email or telephone when it is possible to do so. Studies for which missing or inadequate data cannot be obtained will be excluded from the analysis.

#### 2.3.7 Assessment of heterogeneity

The Mantel-Haenszel chi-square test and the *I*-squared (*I*^2^) statistic of homogeneity will be used to assess study heterogeneity. Studies will be determined to be unsuitable for inclusion in the analysis when the assessment of heterogeneity yields a low *P* value (*P* < .10) together with a high *I*^2^ value, which indicates significant heterogeneity.

#### 2.3.8 Reporting bias assessment

If more than 10 studies are incorporated in the data synthesis, then the reporting bias will be assessed using a funnel plot. Egger's test will be used to evaluate the funnel plots. If Egger's test indicates an asymmetrical funnel plot or *P* < .10, then the data synthesis will be determined to have publication bias. In contrast, a symmetrical funnel plot indicates the presence of no publication bias.

#### 2.3.9 Data synthesis

RevMan software will be used to pool 2 or more eligible studies with low heterogeneity (*P* ≥ .1 and *I*^2^ < 50%) for the meta-analysis. A fixed-effects model will be used to analyze the results of the data synthesis. If *P* < .1 or *I*^2^ ≥ 50%, then the data synthesis will be performed with a random-effects model. If the meta-analysis is infeasible due to considerable methodological and clinical heterogeneity, then a narrative summary will be provided.

#### 2.3.10 Subgroup analysis

If there is significant heterogeneity and a sufficient number of studies are included, then a subgroup analysis will be performed based on the characteristics of the participants, disease stage, interventions, and controls. This analysis will compare the effects of the treatment among subgroups and assess potential reasons for heterogeneity.

#### 2.3.11 Sensitivity analysis

Sensitivity analysis will be performed to explore the quality and robustness of the results of the data synthesis when the outcome analyses involve a high degree of heterogeneity. In this case, the data synthesis will be performed again, but studies with a high risk of bias and small sample size will be excluded. Conclusions must be drawn cautiously from the meta-analysis when inconsistent results are identified by the sensitivity analysis.

#### 2.3.12 Evidence quality evaluation

Evaluation of the quality of evidence for study outcomes will be performed based on the Grading of Recommendations, Assessment, Development, and Evaluation (GRADE) guidelines. Accordingly, the quality of evidence will be assigned one of four grades: high, moderate, low, or very low.

## 3 Discussion

This protocol describes a systematic review and meta-analysis evaluating the effects and safety of warm needling therapy as a treatment for LUTS associated with BPH. A systematic review of acupuncture for BPH by Wei Zhang and the co-authors included data from 8 studies, which were obtained via a database search on September 1, 2016.^[21]^ The results of this analysis showed that patients in the acupuncture group with moderate to severe BPH obtained statistically significant benefit in short-term follow-up endpoints. Although warm needling therapy may be assumed to represent a specific manipulation of acupuncture, the previous systematic review did not include RCTs that evaluated warm needling therapy. A preliminary search yielded more than 20 RCTs that evaluated the effects of warm needling therapy on LUTS associated with BPH. The review described here will provide a clinically relevant evaluation of the current state of evidence regarding the therapeutic efficacy of warm needling therapy for LUTS associated with BPH, which addresses the deficiencies of the previous study and may benefit patients, practitioners and policymakers.

## Acknowledgments

We would like to acknowledge Dr Wei-Guang Li and Dr Hui-Chao Xu at the Department of Urology, Beijing Hospital of Traditional Chinese Medicine, Capital Medical University, Beijing, China, who provided helpful suggestions for the review.

## Author contributions

Tao Zhang and Jing-Qing Sun formulated the study. Tao Zhang drafted the manuscript. Bin Li, Hui-Lin Liu, and Shao-Song Wang planned the search strategy. Fan Zhang, Xin Du, Wei You, and Lian-Cheng Jia reviewed the manuscript and performed revisions. The final version of the manuscript has been read and approved by all authors.

**Conceptualization:** Tao Zhang, Jing-Qing Sun.

**Funding acquisition:** Tao Zhang.

**Investigation:** Fan Zhang, Xin Du, Wei You, Lian-Cheng Jia.

**Methodology:** Bin Li, Hui-Lin Liu, Shao-Song Wang.

**Project administration:** Tao Zhang.

**Supervision:** Bin Li, Hui-Lin Liu, Shao-Song Wang, Jing-Qing Sun.

**Writing – original draft:** Tao Zhang, Jing-Qing Sun.

**Writing – review & editing:** Tao Zhang, Fan Zhang, Xin Du, Wei You, Lian-Cheng Jia, Jing-Qing Sun.
